# On the Puriform Destruction of a Great Part of the Substance of the Brain, without Any Visible Cause

**Published:** 1800-01

**Authors:** 

**Affiliations:** Nantes


### *On the Puriform Destruction of a great Part of the Substance of the Brain, without any visible Cause.* By Cit. Chizeau, *of* Nantes.

[Extracted from the 34th Number of the Recueil Periodique.]

A Child of a very healthy appearance, and whose mother was likewise in good health, had been for four months afflicted with slight indispositions, which were attributed by the mother to the want of milk. The child was in consequence weaned, and fed with new cow's milk, a decoction of pearl barley and rice, and a light pap made of wheat flour previously dried in an oven. About fifteen days afterwards it was seized with a cough, accompanied with uneasiness and vomiting of the pap, mixed with slimy matter. The alvine evacuations were regularly performed, but the child did not sleep sound. At this time Chizeau was consulted, who ordered a change of diet, gentle cathartics, and the use of the syrup of ipecacuanha, which produced scarcely any effect. He had intended to apply leeches, but was prevented by the weak state of the child. A good wet-nurse was procured, but the symptoms still continued. The child became dull, its eyes lost their brilliancy, the eye-lids frequently closed, and it seemed to take no notice of surrounding objects. The pupil was dilated, and the iris did not contract at mid-day; its whole frame was less sensible; convulsions often appeared in the eyes, face, and limbs, and, at length, a complication of the most distressing symptoms. He then prescribed some antispasmodics; and a small quantity of wine, in a diluted state, was given with a view to support the strength of the body. A fortnight afterwards, there appeared on the arm and left hand a light tumour, which much impeded the motion of those parts; the left eye was nearly closed; the child seemed to see indistinctly, and could not suck. The last means employed were were pectoral ſyrups, a little milk, chicken broth, with emollient clyſters, and a ſmall bliſter to the region of the ſtomach: this application was directed with a view to ſtop the hiccough, which ſuddenly increaſed, and much fatigued the child. As little benefit was expected from theſe remedies, the patient died, after languiſhing ſix weeks, at the age of nine months and a half.

On opening the cranium, at the moment of ſeparating the occiput, a quantity of limpid water iſſued forth. The left hemiſphere and ventricle of the brain were perfectly ſound, but the dura and pia mater were thick and inflamed in the part correſponding with the right hemiſphere; the cortical ſubſtance was ſound, and interſperſed with ſpots ſimilar to thoſe of an inflammation; but the greateſt ravages had taken a deeper ſeat: the whole of this hemiſphere was one purulent maſs, without ſmell, and had not the ſmalleſt trace of its natural organization. The tentorium cerebelli appeared changed, and on lifting it up, it was found to contain a quantity of limpid fluid, which iſſued through the foramen ovale, along the medulla oblongata and ſpinalis. The abdomen had alſo undergone ſome change: the ſtomach and the ſmall inteſtines were ſhrivelled; the larger, on the contrary, appeared diſtended and inflamed; in conſequence of which the bladder contained a quantity of urine.

What is the cauſe of ſo many diſeaſes, which do not originate from any blow or external injury?—This is the queſtion addreſſed by Cit. Chizeau to obſervers; an anſwer to which may be of great utility, as will appear by the following details.

*Extract from the Proces-verbal of the Sitting of the 22 Prairial, Seventh Year, containing the Obſervations and Reflections of the Members of the Society, on the foregoing Obſervations.*

A member ſtated, that he knew a citizen, who was the father of four children; the eldeſt of whom, after having enjoyed a good ſtate of health till he was nearly four years of age, fell into a ſtate of languor and debility, which brought him to the grave: this diſeaſe aroſe from hydrocephalus. The ſecond continued in health till he attained the ſame age, when he alſo fell ſick, and died; though bliſters, cauteries, and other appropriate means were employed. When the third child was two years old, a cautery and ſeton, as well as various remedies, were unſucceſsfully tried, and its life could not be preſerved. The fourth child is now three years and a half old; it enjoys good health, but its parents are in the greateſt anxiety.—What means ought to be adopted to prevent the danger which threatens this child?

Another member knew a healthy father and mother, who loſt three three children successively in a similar manner: he likewise wished to obtain some useful hints from the deliberations of the Society.

Several persons gave their opinion. One of the Members observed, that the disease in question was endemic at Geneva; but the causes and prophylactic treatment of it were not discovered. Another Member supposed it to proceed from an organic defect. It was unanimously proposed to keep the belly at liberty; to produce an abundant perspiration; to employ the actual cautery; to apply the *moxa* to both sides of the neck; to remove children threatened with this disease to a pure and dry country air, and cover their head entirely with a blister. The two last means had been unsuccessfully tried on some of the patients above mentioned.

There are two distinct species of dropsy of the brain; one is produced by relaxation; the other by an inflammation often occasioned by the effort of nature in dentition; or sometimes in consequence of metastases from *tinea capitis*. The former cause brings on a serous discharge; the latter produces suppuration. In the first case, diaphoretics, dry frictions, depuratives, tonics, and cauteries, are indicated; in the second, the antiphlogistic treatment, leeches, and revulsive remedies are proper.

